# Colds

**Published:** 1883-04

**Authors:** 


### HALL'S JOURNAL OF HEALTH AND MISCELLANY.

MONTHLY.

E. H. GIBBS, A. M., M. D., EDITOR.

FOUNDED IN 1854.

### COLDS.

THE old proverb, "Feed a cold and starve a fever," is a mischievous fallacy; for with cold there is always fever; and the cold cannot get well until the fever subsides, and the fever will not readily subside while the food supply is kept up.

Warmth and abstinence are the best remedies for colds, and should be applied promptly to insure favorable results.

Warmth keeps the pores of the skin open, thus relieving the system of the surplus secretions which oppress it; abstinence cuts off the supply of material which makes phlegm, which would otherwise have to be coughed up, thus rasping the throat and irritating the air passages, if not laying the foundation for diseases of the throat and lungs, which are liable to endure to the close of life.

The moment a person is satisfied he has taken cold let him do four things:

FIRST.—Take a foot-bath as warm as it can be comfortably borne, letting the feet remain in the water ten to fifteen minutes.

SECOND.—Go to bed in a comfortable room, and cover up well.

THIRD.—Eat nothing.

FOURTH.—Drink as much cold water as he wants, and as much herb tea as he can conveniently; in three cases out of four he will be well in thirty-six hours; but as the system is sensitive for a while after the treatment, care should be taken not to contract a new cold.

If a cold is neglected for two days, it will generally run its course of about two weeks in spite of any treatment; the cough does not usually commence until a day or two after the cold is taken, thus giving the cold three or four days to become fixed in the system; and just here is the starting point of a multitude of diseases. The secretions are checked, the natural functions of the body are weakened, and there is little resistance to disease; hence the liability to rheumatism, congestions that end in consumption, and that disease so fatal after middle age—pneumonia.

When a cold becomes seated, then the only safe plan is to place yourself on the "sick list," and remain in the house until health is completely restored.

